# Impact of commune level social inequalities on time to diagnosis and follow-up in women with high-grade cervical lesions in Isère, France: Evidence from the French cervical cancer screening program

**DOI:** 10.1016/j.pmedr.2026.103396

**Published:** 2026-01-31

**Authors:** Christian Balamou, Karine Zysman, Christelle M. Rodrigue, Arnaud Seigneurin

**Affiliations:** a Université Grenoble Alpes – UGA - Adresse CS 40700 - 38058, Grenoble, France; b Translational Innovation in Medicine and Complexity / Recherche Translationnelle et Innovation en Médecine et Complexité, UMR 5525 – Laboratoire 1043049, Domaine de la Merci, 38706 La Tronche, France; c Centre Régional de Coordination des Dépistages des Cancers en Auvergne-Rhône-Alpes, Site de l'Ain, 01000 Bourg-en-Bresse, France; d Centre Régional de Coordination des Dépistages des Cancers en Auvergne-Rhône-Alpes, Site de l'Isère, 38240 Meylan, France; e Univ. Grenoble Alpes, CNRS, TIMC, CHU Grenoble Alpes, 38000 Grenoble, France; f Registre du Cancer de l'Isère, Centre Hospitalier Universitaire de Grenoble, BP 217, Pavillon E, 38043, Grenoble Cedex 9, France

**Keywords:** High-grade intraepithelial lesions, Cervical cancer screening, Uterine cervical neoplasms, Cervical pap smear, Time to diagnosis, Women's health

## Abstract

**Objective:**

To assess the impact of commune level social inequality characteristics on delays in access to cervical cancer screening, diagnosis, and initial treatment of high-grade cervical intraepithelial lesions.

**Methods:**

We conducted a retrospective cross-sectional study among asymptomatic women living in Isère (department in France) who had undergone at least one cervico-uterine smear between January 1, 2010 and December 31, 2018. Patients with positive screening results were referred for further diagnostic procedures. Social disparities were assessed using the French Deprivation Index.

**Results:**

3060 cases of high-grade cervical intraepithelial lesions with complete diagnosis dates were included in the analysis. Women from the most socioeconomically disadvantaged quintiles experienced significantly longer diagnostic delays than women from more advantaged groups (quintile 2: 0.72 [95% confidence interval: 0.58, 0.91]; quintile 3: 0.76 [95% confidence interval: 0.60, 0.98]). No significant association was found between socioeconomic factors and delays in treatment initiation.

**Conclusions:**

Our findings indicate significantly longer times to diagnostic procedures for women from socioeconomically disadvantaged backgrounds and those with low-grade cytological abnormalities. Given the potential progression to invasive cervical cancer, it may be advisable to allocate specific resources to ensure appropriate follow-up for women with low-grade cytological lesions, particularly those residing in socioeconomically disadvantaged areas.

## Highlights

• How social inequality affects delays in access to diagnosis of cervical lesions.
• Women from the most disadvantaged quintiles experienced longer delays to diagnosis.
• No significant link found between social inequality and time to treatment.
• Over half of women received high-grade cervical lesion diagnoses within two months.
• Allocate resources to follow up women with low-grade cytology in underserved areas.

## 1 Introduction

Global cancer statistics indicate that cervical cancer is the fourth most common cancer in women in terms of both incidence and mortality, with an estimated 660,000 new cases and 350,000 deaths worldwide in 2022 (Bray et al., 2024). A wide range of risk factors has been associated with the development of cervical cancer, among which infection by high-risk human papillomavirus (HPV) types remains the primary cause (CIRC, 2023; Perkins et al., 2023; Tewari, 2025).

Several studies have highlighted the negative impact of sociodemographic and economic factors on HPV vaccination uptake and participation in cervical cancer screening (CCS) programs (Cetina-Pérez et al., 2024; Elit et al., 2013; Elit et al., 2012; Wearn and Shepherd, 2024). In France, similar findings have been reported, showing that both individual and contextual socioeconomic characteristics are associated with lower adherence to CCS recommendations (Audiger et al., 2022; Le Bihan-Benjamin et al., 2023; Traoré et al., 2020). While the relationship between socioeconomic determinants and screening participation is well documented in the French context, few studies have explored the association between these factors and the detection of high-grade cervical intraepithelial lesions (including high-grade squamous intraepithelial lesions and adenocarcinoma in situ - HSIL) of the cervix. Our study was performed to assess the impact of commune level social inequality characteristics on delays in access to CCS, diagnosis, and initial treatment of HSIL.

## 2 Methods

We conducted a retrospective, cross-sectional analytical and descriptive study. The results are reported in accordance with the Strengthening the Reporting of Observational Studies in Epidemiology guidelines (von Elm et al., 2007).

### 2.1 Study design and population

The study included all women living in Isère, a French department in the Auvergne-Rhône-Alpes region, between January 1, 2010 and December 31, 2018. This study period was selected to ensure both data completeness and program stability. In 2019, the French National CCS Program in Isère experienced a temporary interruption related to structural reorganization, the nationwide rollout of the program and the COrona VIrus Disease-19 (COVID-19) pandemic. During this period, the systematic invitation letters were suspended.

The target population consisted of asymptomatic women aged 25–65 years, with no risk factors other than age. Eligible women who had not had a cervical smear reimbursed by the health insurance fund or had no otherwise documentation of screening in the past 3 years were sent personal invitation letters (Hamers et al., 2018) to undergo screening with a conventional cervico-uterine smear. At the time of this initial invitation, no clinical or paraclinical investigations are performed to rule out the presence of HSIL or their early manifestations.

Reimbursement rates for the standard fee (€30 to €40) vary depending on the type of healthcare provider consulted (gynecologist, general practitioner, or midwife), covering either 30% or 70% of the cost. Specialists may apply additional fees, and supplementary health insurance may cover the remaining expenses, subject to specific conditions (ameli, 2025). If the result was normal, the woman was invited for repeat screening 3 years later. In the case of a positive result, the patient was referred for further diagnostic procedures and treatment (Brun et al., 2025).

### 2.2 Variables

The social inequality characteristics of the study population were assessed at the commune level using two indicators:

• The French Deprivation Index (FDep) was developed to provide a geographic indicator of social disadvantage specifically tailored for population-based health studies in France (Rey et al., 2009). In our study, we used the 2015 version of the FDep, which was divided into quintiles (with Quintile-5 [Q5] representing the most deprived population).
• The classification of French municipalities was used to describe and analyze medical deserts in primary care. Bonal et al. conducted a principal component analysis, which, through clustering, identified seven clusters (Bonal et al., 2024).

Abnormal or positive screening test results were classified according to the Bethesda terminology (Nayar and Wilbur, 2015). HSIL were defined according to the World Health Organization classification of tumors of the uterine cervix (Stoler et al., 2014).

Time to screening was defined as the interval between the date of the previous smear and the date of the abnormal screening test.

The histological diagnosis of HSIL was established using specimens obtained by biopsy, curettage, conization, or hysterectomy. Time to diagnosis was defined as the interval between the date of the abnormal screening test and the date of the pathology report from the diagnostic procedure.

The first treatment of HSIL could be surgical (conization or hysterectomy) or non-surgical (laser therapy). The delay before initiating treatment was defined as the interval between the date of HSIL diagnosis and the date of either surgical or laser treatment. All time intervals were calculated using complete dates, including day, month, and year.

Age at the time of the positive test was recorded in categories: 25–29, 30–34, 35–39, 40–44, 45–49, 50–54, 55–59, and ≥ 60 years. Previous screening (prior to the abnormal test) was categorized by the number of prior participations in CCS campaigns in Isère (opportunistic and organized): 0, 1–2, or ≥ 3.

### 2.3 Statistical analysis

**Fig. 1:**
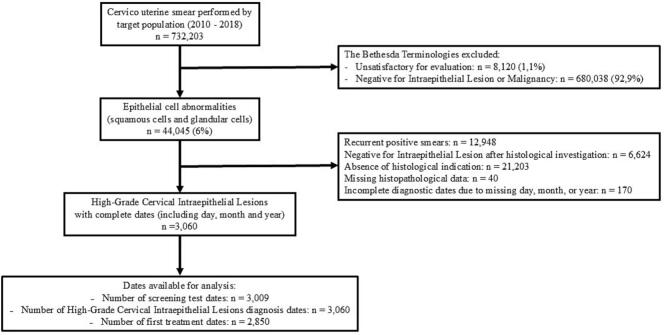
Flow diagram eligibility for high-grade cervical lesions.

The data analyzed were extracted on May 31, 2023, from the Isère management database, which is continuously updated, including during the interruption of the CCS program. Accordingly, sociodemographic and medical information related to HSIL diagnoses and treatment pathways was collected by healthcare professionals and made available to the Isère database (Balamou et al., 2024). We restricted our analysis to HSIL detected in initial positive smears. These lesions underwent standard follow-up from diagnosis through the initiation of first-line treatment. (Fig. 1).

Categorical variables are presented using numbers and percentages. Quantitative variables are presented as medians and interquartile ranges for non-normally distributed variables. For women who received an invitation to participate in screening, time to screening was also measured with consideration of the invitation date.

Time to screening, time to diagnosis, and time to first treatment were analyzed as dependent variables using multivariable logistic regression. The screening threshold was set at 42 months (Balamou et al., 2024). Time to diagnosis was dichotomized at 90 days, in accordance with the U.S. Population-Based Research Optimizing Screening Through Personalized Regimens (PROSPR) consortium (Doubeni et al., 2018). In the absence of established guidance, the median time to first treatment was used as the threshold. Results were expressed as adjusted odds ratios (aORs) with corresponding 95% confidence intervals (CIs).

The HPV vaccine was introduced into the vaccination schedule in France in 2007. We hypothesize that this introduction of the HPV vaccine has not had a protective effect on our study population. Accordingly, vaccination data were not included in our analysis.

The chi-square test was used to compare proportions. Statistical significance was set at *p* ≤ 0.05. Analyses were conducted using R software, version 4.3.1.

## 3 Results

Between January 1, 2010 and December 31, 2018, 732,203 cervico-uterine smears had been performed on 305,940 women. The number of positive smears was 44,045 (6.0%) and several positive smears observed in some women (positive control smears as part of follow-up). The first positive smears observed in each woman was retained: 31,097 cases, which represents 4.2% of all smears. These positive results led to the diagnosis of 3230 cases of HSIL. Only smears with a complete HSIL diagnosis date—including day, month, and year—were included in the analysis, yielding a total of 3060 cases (Fig. 1).

### 3.1 Description of the study population

Table 1 presents the completeness rates of the available dates. Additionally, it presents, for each studied timeframe, the proportion of cases for which it could be calculated, along with the corresponding median and interquartile range. The time to HSIL diagnosis (from the screening test to diagnosis) could be calculated in 98.3% of cases. The women with HSIL received a diagnosis within 2 months (58 days). The time to the first therapeutic proposal (from diagnosis to surgical or laser treatment) was available in 93.1% of cases, with a median of 50 days [27, 83] (Table 1). Additional characteristics of the study population have been previously reported by Balamou et al. (Balamou et al., 2024).

**Table 1:** Clinical characteristics of women with high-grade cervical lesions in the French Cervical Cancer Screening Program in Isère, France (2010–2018).

Characteristics	Number of participants with:		
Dates	Complete dates†	Incomplete dates†	
Previous smear⁎	979 (32.0%)	2081 (68.0%)	
Invitation	1081 (35.3%)	1979 (64.7%)	
Screening test	3009 (98.3%)	51 (1.7%)	
Diagnosis of high-grade cervical intraepithelial lesions	3060 (100%)	–	
*Biopsy*	*2743 (89.6%)*	*317 (10.4%)*	
*Curettage*	*150 (4.9%)*	*2910 (95.1%)*	
*Diagnostic conization*	*283 (9.2%)*	*2777 (90.8%)*	
*Hysterectomy*⁎⁎	*11 (0.4%)*	*3049 (99.6%)*	
Surgical treatment	2751 (89.9%)	309 (10.1%)	
*Conization*	*2701 (88.3%)*	*359 (11.7%)*	
*Hysterectomy*	*132 (4.3%)*	*2928 (95.7%)*	
Laser therapy	147 (4.8%)	2913 (95.2%)	
First treatment of high-grade cervical intraepithelial lesions	2850 (93.1%)	210 (6.9%)	
Types of delays	Number of participants with:		Median [interquartile range]
	complete dates†	incomplete dates†	
Time to screening	959 (31.3%)	2101 (68.7%)	777 [486, 1138]
*Time to screening with invitation*	*223 (7.3%)*	*2837 (92.7%)*	*908 [514, 1410]*
*Time to screening without invitation*	*736 (24.1%)*	*2324 (75.9%)*	*758 [463, 1055]*
Time to diagnosis	3009 (98.3%)	51 (1.7%)	58 [34, 122]
*Time to diagnosis with HSIL and ASC-H*	*1285 (42.0%)*	*1775 (58.0%)*	*46 [30, 75]*
*Time to diagnosis with ASC-US and LSIL*	*1661 (54.2%)*	*1399 (45.8%)*	*76 [42, 217]*
*Time to diagnosis with AGC*	*63 (2.1%)*	*2997 (97.9%)*	*56 [34, 79]*
Time to surgical treatment	2751 (89.9%)	309 (10.1%)	50 [27, 83]
Time to laser therapy	147 (4.8%)	2913 (95.2%)	62 [28, 130]
Time to first treatment	2850 (93.1%)	210 (6.9%)	50 [27, 83]

Data are presented as n (%).

ASC-US: atypical squamous cells of undetermined significance; LSIL: low-grade squamous intraepithelial lesion; HSIL: high-grade squamous intraepithelial lesion; ASC—H: atypical squamous cells that cannot exclude HSIL; AGC: atypical glandular cells.

† Percentages are calculated based on the total number of 3060 cases with complete high-grade cervical intraepithelial lesions diagnosis dates (including day, month, and year).

⁎ The limited number of previous smears may be due to the absence of systematic data collection within the screening program, or the inclusion of women whose screening history was unavailable (e.g., newly arrived residents or women recently enrolled in the program, such as those aged 25 years).

⁎⁎ Hysterectomy performed due to a positive smear associated with uterine pathology (e.g., progressive fibroid, leiomyoma, or myoma with ovarian cancer).

### 3.2 Factors associated with delays

The associations between time to screening, time to diagnosis, time to first treatment, and predictive factors were investigated (Table 2, Table 3, Table 4). Among women diagnosed with HSIL, the multivariate analysis showed that adherence to the recommended screening interval was significantly more frequent in the most disadvantaged FDep quintiles (Q3, Q4, and Q5) than in the reference quintile (Q1) (Q3: aOR 1.48 [95% CI: 1.13, 1.94]; Q4: aOR 1.53 [95% CI: 1.16, 2.02]; Q5: aOR 1.98 [95% CI: 1.29, 3.03]) (Table 2).

**Table 2:** Association between time to screening (≤42 months, yes/no) and predictive factors among women with high-grade cervical lesions in the French Cervical Cancer Screening Program in Isère, France (2010–2018): logistic regression model.

	Descriptive analysis			Bivariate model		Multivariate model	
Characteristics	Screening >42 months *n* = 2270	Screening ≤42 months *n* = 790	*p*-value†	OR	95% CI	aOR	95% CI
FDep			<0.01				
Quintile-1	498 (22.2%)	143 (18.2%)		–	–	–	–
Quintile-2	743 (33.1%)	228 (29.0%)		1.07	0.84, 1.36	1.14	0.88, 1.47
Quintile-3	457 (20.4%)	191 (24.3%)		1.46	1.13, 1.87	1.48	1.13, 1.94
Quintile-4	437 (19.5%)	173 (22.0%)		1.38	1.07, 1.78	1.53	1.16, 2.02
Quintile-5	107 (4.8%)	50 (6.4%)		1.63	1.10, 2.38	1.98	1.29, 3.03
Missing	28	5					
French municipalities classification			0.20				
Clusters 5, 6, and 7 (municipalities generally favored in terms of accessibility to health care, though mitigated in some cases by high needs or a declining supply)	1990 (88.9%)	683 (87.0%)		–	–	–	–
Clusters 3 and 4 (municipalities with poor accessibility to health care)	229 (10.2%)	90 (11.5%)		1.15	0.88, 1.48	1.09	0.82, 1.44
Clusters 1 and 2 (municipalities limited access to specific types of health care professionals)	20 (0.9%)	12 (1.5%)		1.75	0.83, 3.55	1.55	0.68, 3.42
Missing	31	5					
Age (years)			0.60				
25–29	559 (24.6%)	202 (25.6%)		–	–	–	–
30–34	500 (22.0%)	176 (22.3%)		0.97	0.77, 1.23	0.66	0.50, 0.85
35–39	417 (18.4%)	136 (17.2%)		0.90	0.70, 1.16	0.55	0.41, 0.73
40–44	364 (16.0%)	106 (13.4%)		0.81	0.61, 1.05	0.48	0.35, 0.64
45–49	216 (9.5%)	84 (10.6%)		1.08	0.80, 1.45	0.58	0.42, 0.81
50–54	105 (4.6%)	44 (5.6%)		1.16	0.78, 1.70	0.61	0.39, 0.93
55–59	65 (2.9%)	26 (3.3%)		1.11	0.67, 1.77	0.59	0.34, 0.99
≥60	44 (1.9%)	16 (2.0%)		1.01	0.54, 1.79	0.56	0.29, 1.06
Types of positive smears			<0.01				
HSIL / squamous carcinoma	683 (30.1%)	199 (25.2%)		–	–	–	–
ASC-US	576 (25.4%)	237 (30.0%)		1.41	1.14, 1.76	1.20	0.94, 1.52
ASC-H	318 (14.0%)	115 (14.6%)		1.24	0.95, 1.62	1.08	0.81, 1.44
Atypical glandular cells / adenocarcinoma	56 (2.5%)	9 (1.1%)		0.55	0.25, 1.08	0.42	0.19, 0.86
LSIL	637 (28.1%)	230 (29.1%)		1.24	1.00, 1.54	1.17	0.92, 1.48
Number of previous screenings			<0.01				
0	1416 (62.4%)	186 (23.5%)		–	–	–	–
1 or 2	713 (31.4%)	444 (56.2%)		4.74	3.91, 5.76	5.32	4.35, 6.54
≥3	141 (6.2%)	160 (20.3%)		8.64	6.58, 11.4	11.0	8.23, 14.8

†Chi-square test. CI: confidence interval; OR: odds ratio; aOR: adjusted odds ratio; FDep: French Deprivation Index; ASC-US: atypical squamous cells of undetermined significance; LSIL: low-grade squamous intraepithelial lesion; HSIL: high-grade squamous intraepithelial lesion; ASC—H: atypical squamous cells that cannot exclude HSIL. The multivariate model equation, applied to a sample size of 3024 subjects, was: glm(Time to screening (≤42 months, yes/no) ∼ FDep+French municipalities classification+Age at positive smear+Types of positive smears+Number of previous screenings,data,family = “binomial”). The best-fitting model (with and without interaction terms) was selected based on the Akaike Information Criterion. Interaction terms were tested between FDep and French municipalities classification; FDep and age at positive smear; French municipalities classification and age at positive smear.

**Table 3:** Association between time to diagnosis (≤90 days, yes/no) and predictive factors among women with high-grade cervical lesions in the French Cervical Cancer Screening Program in Isère, France (2010–2018): logistic regression model.

	Descriptive analysis			Bivariate model		Multivariate model	
Characteristics	Diagnosis >90 days *n* = 989	Diagnosis ≤90 days *n* = 2020	p-value†	OR	95% CI	aOR	95% CI
FDep			0.03				
Quintile-1	183 (18.6%)	449 (22.5%)		–	–	–	–
Quintile-2	341 (34.7%)	606 (30.4%)		0.72	0.58, 0.90	0.72	0.57, 0.91
Quintile-3	216 (22.0%)	422 (21.2%)		0.80	0.63, 1.01	0.76	0.59, 0.98
Quintile-4	200 (20.3%)	405 (20.3%)		0.83	0.65, 1.05	0.78	0.60, 1.00
Quintile-5	43 (4.4%)	111 (5.6%)		1.05	0.72, 1.57	0.97	0.65, 1.48
Missing	6	27					
French municipalities classification			0.60				
Clusters 5, 6 and 7 (municipalities rather favored in terms of accessibility to health care, but mitigated for some by high needs or declining supply)	875 (89.1%)	1751 (87.9%)		–	–	–	–
Clusters 3 and 4 (municipalities with poor accessibility to health care)	96 (9.8%)	219 (11.0%)		1.14	0.89, 1.47	1.11	0.85, 1.45
Clusters 1 and 2 (municipalities that have difficulty accessing only certain types of health care professionals)	11 (1.1%)	21 (1.1%)		0.95	0.47, 2.06	0.81	0.38, 1.80
Missing	7	29					
Age (years)			<0.01				
25–29	283 (28.6%)	464 (23.0%)		–	–	–	–
30–34	234 (23.7%)	429 (21.2%)		1.12	0.90, 1.39	1.04	0.82, 1.31
35–39	154 (15.6%)	394 (19.5%)		1.56	1.23, 1.98	1.42	1.10, 1.82
40–44	142 (14.4%)	316 (15.6%)		1.36	1.06, 1.74	1.25	0.96, 1.62
45–49	77 (7.8%)	221 (10.9%)		1.75	1.30, 2.37	1.57	1.15, 2.17
50–54	45 (4.6%)	101 (5.0%)		1.37	0.94, 2.02	1.26	0.84, 1.91
55–59	32 (3.2%)	57 (2.8%)		1.09	0.69, 1.73	0.90	0.55, 1.47
≥60	22 (2.2%)	38 (1.9%)		1.05	0.62, 1.84	0.89	0.50, 1.60
Types of positive smears			<0.01				
HSIL / squamous carcinoma	151 (15.3%)	709 (35.1%)		–	–	–	–
ASC-US	384 (38.8%)	422 (20.9%)		0.23	0.19, 0.29	0.23	0.19, 0.29
ASC-H	95 (9.6%)	330 (16.3%)		0.74	0.56, 0.99	0.74	0.55, 0.99
Atypical glandular cells / adenocarcinoma	15 (1.5%)	48 (2.4%)		0.68	0.38, 1.29	0.68	0.37, 1.28
LSIL	344 (34.8%)	511 (25.3%)		0.32	0.25, 0.39	0.32	0.25, 0.40
Number of previous screenings			0.40				
0	520 (52.6%)	1051 (52.0%)		–	–	–	–
1 or 2	382 (38.6%)	759 (37.6%)		0.98	0.84, 1.16	1.02	0.86, 1.21
≥3	87 (8.8%)	210 (10.4%)		1.19	0.91, 1.57	1.20	0.90, 1.61

†Chi-square test. CI: confidence interval; OR: odds ratio; aOR: adjusted odds ratio; FDep: French Deprivation Index; ASC-US: atypical squamous cells of undetermined significance; LSIL: low-grade squamous intraepithelial lesion; HSIL: high-grade squamous intraepithelial lesion; ASC—H: atypical squamous cells that cannot exclude HSIL. The multivariate model equation, applied to a sample size of 2973 subjects, was: glm(Time to diagnosis (≤90 days, yes/no) ∼ FDep+French municipalities classification+Age at positive smear+Types of positive smears+Number of previous screenings,data,family = “binomial”). The best-fitting model (with and without interaction terms) was selected based on the Akaike Information Criterion. Interaction terms were tested between FDep and French municipalities classification; FDep and age at positive smear; French municipalities classification and age at positive smear.

**Table 4:** Association between time to first treatment (≤50 days, yes/no) and predictive factors among women with high-grade cervical lesions in the French Cervical Cancer Screening Program in Isère, France (2010–2018): logistic regression model.

	Descriptive analysis			Bivariate model		Multivariate model	
Characteristics	Treatment >50 days *n* = 1405	Treatment ≤50 days *n* = 1445	p-value†	OR	95% CI	aOR	95% CI
FDep			0.30				
Quintile-1	302 (21.8%)	299 (20.9%)		–	–	–	–
Quintile-2	468 (33.8%)	450 (31.4%)		0.97	0.79, 1.19	1.01	0.82, 1.24
Quintile-3	288 (20.8%)	316 (22.0%)		1.11	0.88, 1.39	1.14	0.91, 1.44
Quintile-4	269 (19.4%)	290 (20.2%)		1.09	0.86, 1.37	1.11	0.88, 1.40
Quintile-5	57 (4.1%)	79 (5.5%)		1.40	0.96, 2.05	1.42	0.97, 2.08
Missing	21	11					
French municipalities classification			0.05				
Clusters 5, 6 and 7 (municipalities rather favored in terms of accessibility to health care, but mitigated for some by high needs or declining supply)	1234 (89.2%)	1257 (87.8%)		–	–	–	–
Clusters 3 and 4 (municipalities with poor accessibility to health care)	142 (10.3%)	155 (10.8%)		1.07	0.84, 1.36	1.07	0.84, 1.36
Clusters 1 and 2 (municipalities with difficulty accessing only certain types of health care professionals)	7 (0.5%)	20 (1.4%)		2.80	1.24, 7.17	2.91	1.27, 7.50
Missing	22	13					
Age (years)			0.06				
25–29	372 (26.5%)	322 (22.3%)		–	–	–	–
30–34	320 (22.8%)	305 (21.1%)		1.10	0.89, 1.37	1.11	0.89, 1.38
35–39	234 (16.7%)	282 (19.5%)		1.39	1.11, 1.75	1.43	1.13, 1.82
40–44	219 (15.6%)	225 (15.6%)		1.19	0.94, 1.51	1.20	0.94, 1.53
45–49	124 (8.8%)	157 (10.9%)		1.46	1.11, 1.93	1.47	1.11, 1.97
50–54	70 (5.0%)	72 (5.0%)		1.19	0.83, 1.71	1.27	0.88, 1.85
55–59	41 (2.9%)	49 (3.4%)		1.38	0.89, 2.15	1.39	0.89, 2.18
≥60	25 (1.8%)	33 (2.3%)		1.52	0.89, 2.64	1.57	0.91, 2.74
Types of positive smears			0.04				
HSIL / squamous carcinoma	382 (27.2%)	463 (32.0%)		–	–	–	–
ASC-US	399 (28.4%)	360 (24.9%)		0.74	0.61, 0.91	0.78	0.64, 0.95
ASC-H	197 (14.0%)	208 (14.4%)		0.87	0.69, 1.10	0.89	0.70, 1.13
Atypical glandular cells / adenocarcinoma	30 (2.1%)	33 (2.3%)		0.91	0.54, 1.52	0.90	0.53, 1.51
LSIL	397 (28.3%)	381 (26.4%)		0.79	0.65, 0.96	0.83	0.68, 1.01
Number of previous screenings			0.60				
0	706 (50.2%)	754 (52.2%)		–	–	–	–
1 or 2	554 (39.4%)	550 (38.1%)		0.93	0.79, 1.09	0.89	0.76, 1.05
≥3	145 (10.3%)	141 (9.8%)		0.91	0.71, 1.17	0.85	0.65, 1.11

†Chi-square test. CI: confidence interval; OR: odds ratio; aOR: adjusted odds ratio; FDep: French Deprivation Index; ASC-US: atypical squamous cells of undetermined significance; LSIL: low-grade squamous intraepithelial lesion; HSIL: high-grade squamous intraepithelial lesion; ASC—H: atypical squamous cells that cannot exclude HSIL. The multivariate model equation, applied to a sample size of 2815 subjects, was: glm(Time to first treatment (≤50 days, yes/no) ∼ FDep+French municipalities classification+Age at positive smear+Types of positive smears+Number of previous screenings,data,family = “binomial”). The best-fitting model (with and without interaction terms) was selected based on the Akaike Information Criterion. Interaction terms were tested between FDep and French municipalities classification; FDep and age at positive smear; French municipalities classification and age at positive smear.

In the multivariate analysis (Table 3), the time to diagnosis was significantly longer in Q2 (0.72 [95% CI: 0.58, 0.91]) and Q3 (0.76 [95% CI: 0.60, 0.98]) than in the reference quintile (Q1). Similarly, compared with high-grade squamous intraepithelial lesion (HSIL) cytology, atypical squamous cells that cannot exclude HSIL (ASC—H) (0.73 [95% CI: 0.55, 0.98]), ASC- of undetermined significance (ASC-US) (0.73 [95% CI: 0.55, 0.98]), and low-grade squamous intraepithelial lesion (LSIL) (0.32 [95% CI: 0.25, 0.40]) results were associated with longer diagnostic delays (Table 3).

The time to the first therapeutic proposal (laser or surgery) varied according to age at positive smear and the types of positive smears (Table 4). Compared with HSIL smears, ASC-US smears (0.78 [95% CI: 0.64, 0.95]) were associated with longer delays to the first therapeutic proposal (i.e., beyond the median). Among individuals who received surgical treatment only, the time to treatment initiation was significantly shorter in quintile Q5 (1.48 [95% CI: 1.01, 2.18]) compared with the reference quintile (Q1).

## 4 Discussion

This study assessed the time to diagnosis among 3060 cases of HSIL. We found that more than half of the women received a diagnosis within 2 months. However, socioeconomic disparities—assessed at the commune level—appeared to influence this timeline. Specifically, women from the most socioeconomically disadvantaged quintiles experienced significantly longer delays to diagnosis than women from more advantaged groups. Interestingly, our results did not show a significant association between social inequality determinants and delays in access to treatment.

Our results show that access to CCS takes longer for women who respond to invitation letters from the organized screening program than for women who initiate screening spontaneously. Previous French studies suggest that offering free screening serves as a facilitator and may help reduce delays in access to care (Audiger et al., 2022). In our multivariate analysis, we observed that women residing in the most socioeconomically disadvantaged quintiles of the FDep showed better adherence to screening access recommendations than those from more advantaged quintiles. Although these findings may appear counterintuitive, they can be interpreted in light of improved compliance observed in certain populations with limited health literacy or greater distance from the healthcare system, once the relevance of screening has been understood and accepted. Several studies support this hypothesis, reporting satisfactory adherence to recommendations among disadvantaged populations when targeted by interventions tailored to their level of health literacy and sociocultural context (Balamou et al., 2023; Cazorla et al., 2025). Similarly, Ferreira et al. showed, in a randomized controlled trial, that an intervention targeting both physicians and patients, including reminders about screening and health literacy, led to a greater improvement (*p* < 0.01) in screening participation among patients with low health literacy compared with the control group (Ferreira et al., 2005).

The available evidence on the optimal timing of diagnostic testing is of very low quality primarily because of the absence of published empirical studies (Doubeni et al., 2018). Nevertheless, it is generally accepted that such investigations should be conducted as promptly as possible. In our study, more than half of the women diagnosed with HSIL underwent diagnostic evaluation within 2 months, with a median time of 58 days. This median interval was shorter for high-grade cytology results (46 days) and longer for ASC-US and LSIL (76 days). These findings align with the recommendations from the PROSPR Consortium, which advocates performing colposcopy within 30 days for suspected invasive disease, within 60 days for high-grade cytology, and within 90 days for women with lower-grade cytology (Doubeni et al., 2018). Additionally, Kupets et al. (Kupets and Paszat, 2011) reported median wait times for colposcopy in Ontario, Canada during the period 2000–2005, ranging from 24 to 39 days for cytology suggestive of malignancy or cancer, 67 days for HSIL, 80 days for ASC—H, and 108 days for atypical glandular cells.

Our multivariate analyses showed that the time to diagnosis of HSIL was significantly longer among women from socioeconomically disadvantaged backgrounds and in cases of low-grade cytological abnormalities. We hypothesize that the prolonged time to diagnosis access observed among women from socioeconomically disadvantaged backgrounds may result from a limited access to colposcopists in disadvantaged areas; structural constraints, such as mobility issues, that may restrict the ability of women in these areas; and the cost of specialist consultations, which can be further increased by additional fees, representing a significant financial barrier for low-income populations.

These findings are consistent with previous studies examining the influence of sociodemographic and socioeconomic factors on the quality of follow-up after the detection of abnormal cervical smears (Doubeni et al., 2018; Elit et al., 2013; Elit et al., 2012; Tabnak et al., 2010; Traoré et al., 2020). Elit et al. (Elit et al., 2013; Elit et al., 2012) reported lower follow-up quality for both low- and high-grade abnormalities among women living in low-income neighborhoods than among women in higher-income areas.

The extended time to diagnosis observed in women with ASC-US or LSIL cytology can be attributed to the clinical guidelines in effect at the time of the study, which did not systematically recommend immediate diagnostic procedures—such as colposcopy—for these types of abnormalities, unlike for high-grade cytological lesions (INCa, 2016). Specifically, in the case of ASC-US, guidelines recommended high-risk HPV testing or, for women under 30 years, reflex dual immunostaining with p16/Ki67 as an alternative to HPV testing. For LSIL cytology, in the absence of access to colposcopy or p16/Ki67 dual staining, repeat cytology at 12 months followed by another at 24 months was advised. A second abnormal cytology would then necessitate colposcopy. The most recent update to the French National Cancer Institute (*Institut national du cancer - INCa*) guidelines (2025) now recommends colposcopy as the first-line management for ASC-US and LSIL in women aged ≥30 years (Brun et al., 2025).

It is generally accepted that timely treatment following a diagnosis of HSIL is essential to prevent progression to invasive cervical cancer (INCa, 2016; Kupets and Paszat, 2011). We did not identify any specific recommendations in the literature regarding the optimal timeframe for the management of abnormal cervical smears. The term most commonly used for high-grade and related cytological abnormalities is “immediate treatment.” In our study, the median time to first treatment after HSIL diagnosis was 50 days [27, 83]. This finding highlights the importance of ensuring prompt and organized management of diagnosed patients—not only to prevent lesion progression but also to reduce treatment-related costs (Doubeni et al., 2018; Frederiksen et al., 2012). Except for ASC-US cytological results—which were associated with significantly longer delays in access to HSIL treatment—none of the other socioeconomic factors assessed in our study had a significant impact on treatment delays. The fact that the management of HSIL is primarily hospital-based may explain the lack of a significant association between treatment delays and social inequality determinants assessed at the commune level. Indeed, hospitals can provide targeted support for women facing social or financial hardship, particularly by offering free or fully covered medical services. In the French health care system, access to diagnostic procedures and treatments is independent of socioeconomic status. These services are reimbursed by National Health Insurance according to standard reimbursement rates (70–80% depending on the procedure), and supplementary health insurance or France's government-funded complementary health insurance for low-income individuals (*Complémentaire santé solidaire*) may cover the remaining expenses, subject to specific conditions (ameli, 2025). In 2013, in France, de Rycke et al. (de Rycke et al., 2020) estimated the total cost of hospital stays for the 34,067 women hospitalized with a diagnosis of SIL at €41,267,068, or a mean cost of €1211 per woman. Seventy-six percent of the total cost arose from stays with an HSIL diagnosis.

As for the strengths of our study, the quality of the database enabled the identification and tracking of 3060 smears with a complete HSIL diagnosis date. Moreover, the screening structure in Isère has been organizing CCS since 1990 (Bolla et al., 1996), achieving a triennial coverage rate of 70.1% for the period 2019–2021 (Santé publique France, 2023). Three main limitations should be taken into consideration when interpreting these findings. First, the absence of individual-level socioeconomic data led to the use of aggregated indicators at the commune level, which were developed to provide a geographic measure of social disadvantage specifically tailored for population-based health studies in France. Second, we chose to focus on HSIL detected in initial positive smears and did not analyze the diagnosis of HSIL lesions identified during follow-up. In addition, we excluded smears for which the HSIL diagnosis date was incomplete. This methodological decision regarding the study population, combined with the screening and diagnostic methods used, resulted in a reduced number of HSIL cases included in the analysis. Third, the presence of missing data is an inherent consequence of retrospective studies but does not compromise the validity of our results.

## 5 Conclusion

To our knowledge, this is the first French study to evaluate the impact of socioeconomic determinants on diagnostic delays among women with high-grade cervical cytological abnormalities, incorporating an analysis of social inequality determinants at the commune level.

The data presented in this study were collected before the COVID-19 pandemic and may serve as a reference point for defining priorities in the follow-up of women with high-grade cytological abnormalities. These findings may also inform the development of strategies aimed at reducing inequalities in access to screening, diagnosis, and treatment of cervical HSIL.

## Disclosure of ethical compliance

Before analysis, all data were anonymised. The screening database had a favourable opinion from the institution that oversees the ethics of data collection (‘Commission Nationale de l'Informatique et des Libertés’) (Journal Officiel de la République Française, 2020).

## Disclosure of funding and conflicts of interest.

The authors declare that this research did not receive any specific grant from funding agencies in the public, commercial, or not-for-profit sectors.

## CRediT authorship contribution statement

**Christian Balamou:** Writing – review & editing, Writing – original draft, Visualization, Project administration, Methodology, Formal analysis, Data curation, Conceptualization. **Karine Zysman:** Writing – review & editing, Visualization, Methodology, Formal analysis, Data curation. **Christelle M. Rodrigue:** Writing – review & editing, Visualization, Methodology, Formal analysis. **Arnaud Seigneurin:** Writing – review & editing, Validation, Supervision, Methodology, Formal analysis, Conceptualization.

## Consent for publication

All authors consent for publication in this journal.

## Declaration of competing interest

The authors declare that they have no known competing financial interests or personal relationships that could have appeared to influence the work reported in this paper.

## Data Availability

Under current French legislation, ethical and regulatory restrictions prevent us from sharing individualized participant data, especially sensitive or confidential information (JORF, 2020).
